# Identification of proteins associated with development of psoriatic arthritis in peripheral blood mononuclear cells: a quantitative iTRAQ-based proteomics study

**DOI:** 10.1186/s12967-021-03006-x

**Published:** 2021-08-03

**Authors:** Jie Zhu, Ling Han, Ruilai Liu, Zhenghua Zhang, Qiong Huang, Xu Fang, Ke Yang, Guiqin Huang, Zhizhong Zheng, Nikhil Yawalkar, Hui Deng, Kexiang Yan

**Affiliations:** 1 Department of Dermatology, Huashan Hospital, Fudan University, Shanghai, China; 2 Department of Laboratory Medicine, Huashan Hospital, Fudan University, Shanghai, China; 3 Department of Information, Huashan Hospital, Fudan University, Shanghai, China; 4 Department of Dermatology, Inselspital, Bern University Hospital, University of Bern, Bern, Switzerland; 5 Department of Dermatology, Shanghai Jiaotong University Affiliated Sixth People’s Hospital, Shanghai, China

**Keywords:** Psoriatic arthritis, Proteomics, iTRAQ, Peripheral blood, Biomarkers

## Abstract

**Background:**

Biomarkers for distinguishing psoriatic arthritis (PsA) from psoriasis without arthritis (PsO) are still lacking.

**Methods:**

We applied isobaric tags for relative and absolute quantification (iTRAQ) and LC–MS/MS to analyze the proteome profile of peripheral blood mononuclear cells (PBMCs) collected from patients with PsO, patients with PsA, and healthy controls. Bioinformatics analysis and western blotting were performed to identify and validate differentially expressed proteins.

**Results:**

We identified 389, 199, 291, and 60 significantly differentially expressed proteins (adj.p < 0.05) in the comparison of all psoriatic patients versus healthy controls, PsO group versus healthy controls, PsA group versus healthy controls, and PsA group versus PsO group, respectively. Among these proteins, 14 proteins may represent promising biomarkers for PsA: SIRT2, NAA50, ARF6, ADPRHL2, SF3B6, SH3KBP1, UBA3, SCP2, RPS5, NUDT5, NCBP1, SYNE1, NDUFB7, HTATSF1. Furthermore, western blotting confirmed that SIRT2 expression was significantly higher in PBMCs from PsA patients than PsO and healthy controls, and was negatively correlated with the phosphorylation of p38 mitogen-activated protein kinase (p-p38MAPK; p = 0.006, r = − 0.582).

**Conclusions:**

This pilot study provided a broad characterization of the proteome of PBMCs in PsA as compared to PsO and healthy controls, which may help to provide prospective strategies for PsA diagnosis.

**Supplementary Information:**

The online version contains supplementary material available at 10.1186/s12967-021-03006-x.

## Background

Psoriasis (PsO) is a common immune-mediated inflammatory disease mediated by dendritic and T cells, as key cell types, and type I interferons (TNF-α, IL-17, and IL-23), as key inflammatory cytokines [1]. Approximately 30% of patients with PsO finally develop psoriatic arthritis (PsA) [2], and they may experience a more significant reduction in quality of life [3]. As many as 29% of PsO patients attending dermatology practices might have undiagnosed PsA, and the diagnosis of early PsA remains a challenge for dermatologists [4]. Since a diagnostic delay of > 6 months may contribute to poor functional and radiographic outcomes, it is critical to detect biomarkers that can facilitate the diagnosis of PsA and predict therapeutic outcomes.

Almost 50% of PsA patients have a positive family history. In comparison to PsO, a cohort of PsA patients showed considerable difference in their predominant HLA alleles [5], indicating that genetics plays a major role in the predisposition to develop PsA. Despite a well-defined genetic cause, there is a growing consensus that because of splicing and protein modifications, systems-level approaches, including the analysis of proteomes, are now required for a more comprehensive understanding of the causes of PsA [6].

Proteomics, as the gene products, has been continuously developed for simultaneous identification and quantification of proteins associated with psoriasis and psoriatic arthritis, especially in the detection of various diagnostic markers for distinguishing PsA from PsO. In 2015, Cretu et al. [7]. first applied quantitative tandem mass-spectrometry to compare PsO and PsA; they identified 47 proteins that were elevated in the skin of 10 patients with PsA compared to that of 10 patients with PsO. Then, serum concentration of CXCL10, measured by Luminex assay, was reported to increase significantly after conversions from PsO to PsA [4] while Reindl et al. demonstrated that only the combination of cartilage oligomeric matrix protein (COMP) together with haptoglobin (HP) in plasma appeared to be appropriate for diagnosing PsA [8]. Furthermore, Matsuura et al. applied MALDI-TOF MS and Triple-TOF MS/MS to serum peptide profiles and found that the ion intensities of TMSB4X protein-derived peptides with 4964 m/z (p4964) and 4979 m/z (p4979) were 1.95- and 1.67-fold lower in the psoriatic arthritis group than their respective ion intensities in the psoriasis vulgaris group [9].

Peripheral blood mononuclear cell (PBMCs) are suitable samples for searching for biomarkers with proteomic techniques since they are easy to obtain and more stable than free-circulating plasma or serum. Nevertheless, the proteome profiling of PBMCs in patients with PsO and those with PsA remains to be elucidated. In the present study, as part of a relative and absolute quantitation (iTRAQ)-based quantitative proteomic approach, isobaric tags were employed to measure proteome changes of PBMCs in PsO and PsA groups, as well as healthy controls. The differentially expressed proteins were further analyzed by bioinformatics analysis and validated by Western blot.

## Methods

### Subjects

**Fig. 1:**
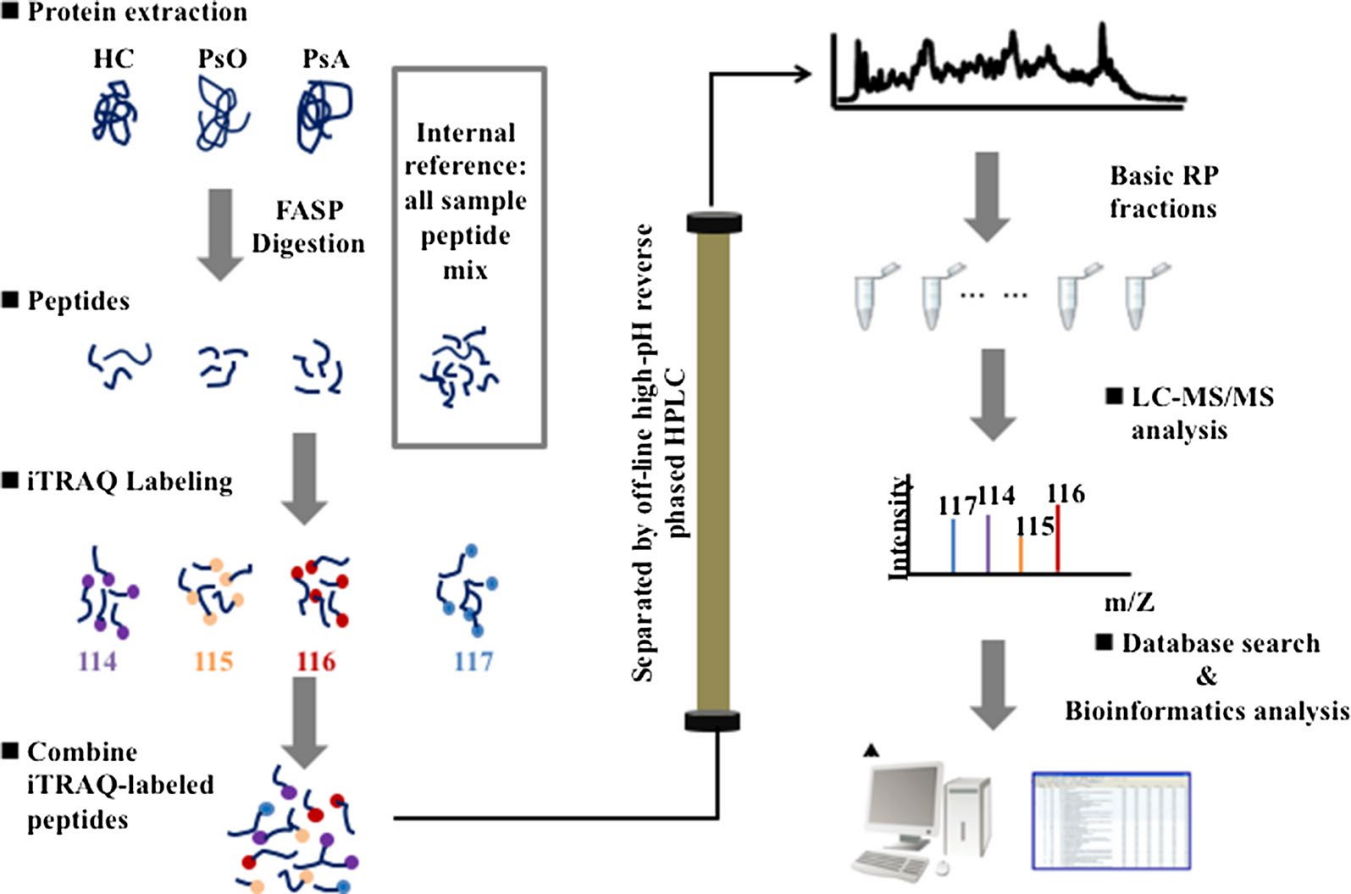
Experimental workflow for iTRAQ labeling proteome analysis. Equal amounts of proteins from 12 samples were digested with trypsin. The resultant peptides were subjected to iTRAQ labeling, HPRP fractionation, and subsequent LC–MS/MS analysis

A total of 33 blood samples were collected, including 12 samples for proteomics analysis (PsO, n = 4; PsA, n = 4; healthy controls (HC), n = 4), and 21 samples for western blotting analysis (PsO, n = 8; PsA, n = 8; HC, n = 5) at the Huashan Hospital, Fudan University. The diagnosis of psoriasis was based on typical clinical and/or histopathological criteria. PsA patients had psoriasis and satisfied the CASPAR classification criteria [10]. Healthy controls were sex-and age-matched with patients. The study was conducted in accordance with the Declaration of Helsinki. The Medical Ethics Committee of Huashan Hospital, Fudan University, reviewed and approved the protocol, and all participants provided written informed consent. 15 ml of peripheral venous blood were collected by venipuncture into EDTA-coated tubes. The peripheral blood mononuclear cells (PBMCs) were isolated via density-gradient centrifugation method using Lymphoprep™ (LMP, Stemcell Technologies, Cambridge, UK) according to the manufacturer’s instructions. All PBMCs samples were frozen in liquid nitrogen and stored at − 80 °C. The workflow is shown in Fig. 1. More detailed procedures on the sample preparation, iTRAQ Labeling, High pH reverse phase fractionation (HPRP), and LC–MS/MS analysis could be found in Additional file 5.

### Western blotting

21 PBMCs samples for western blotting analysis were washed with cold PBS and lysed in RIPA lysis buffer (Beyotime, China) supplemented with protease and phosphatase inhibitors and PMSF (Beyotime, China). Protein concentrations of samples were determined by the BCA assay (Beyotime, China). The protein (40 μg/lane) were separated by 10% SDS-PAGE and transferred onto polyvinylidene fluoride (PVDF) membrane (Millipore, USA). Membranes were blocking with 5% skim milk for 2 h and then incubated at 4 °C overnight with the different primary antibodies respectively. The antibodies were rabbit anti-SIRT2 (ab211033, 1:2000 dilutions; abcam), rabbit anti-phospho-P38 (# 4511, 1:1000 dilutions; CST), rabbit anti- GAPDH (# 5174, 1:1000 dilutions, CST, set as additional loading controls). Membranes were then incubated with the secondary fluorescent antibody (Beyotime, China) at 37 °C for 1 h. The blotting bands were analyzed using the Odyssey Infrared Imaging system (LI-COR, USA).

### Statistical analysis

The *limma* method was performed to detect the difference of PBMCs proteome profiling among patients with PsO, patients with PsA, and healthy controls. *Limma* is a highly recommended tool for comparative proteomics analysis of data sets with low replicate numbers, which not only outperforms the standard t test in terms of number of differentially regulated features but also maintains the right estimate for false positives for sufficiently large feature numbers. Protein fold changes (on log scale) were computed and differential expression P values corrected for multiple testing using the Benjamini-Hochberg [false discovery rate (FDR)] method. Proteins with an adjusted P value of < 0.05 were considered as significantly differentially expressed between groups. All computations were performed in R statistical programming environment (version 3.6.3). Kyoto Encyclopedia of Genes and Genomes (KEGG) signaling pathway analysis was performed using DAVID Bioinformatics Resources version 6.8. Protein–protein interaction analysis was performed using STRING Version 11.0 (https://string-db.org/) and GeneMania (Version v3.5.2, https://genemania.org) [11]. The score of minimum required interaction in STRING was medium confidence (0.400).

## Results

### Patient characteristics

As shown in Additional file 1: Table S1, we recruited eight patients with psoriasis (four with PsO and four with PsA, five men, three women; mean age 55.5 years, range 36–86 years) and four age- sex-matched healthy controls for proteomics analysis. The average PASI score was 14.3 (range 9.3–22.5), and the average BSA score was 33.1 (range 13.0–53.0). Two patients had a history of hypertension; one patient had a history of diabetes mellitus; two patients had a history of hyperlipidemia; and four patients reported a history of smoking. As shown in Additional file 2: Table S2, 16 patients (8with PsO and 8 with PsA) and age-sex-matched healthy controls was recruited for western blotting validation of SIRT2 protein expression (Additional file 3: Figure S1).

### Potential biomarkers for psoriatic arthritis

**Fig. 2:**
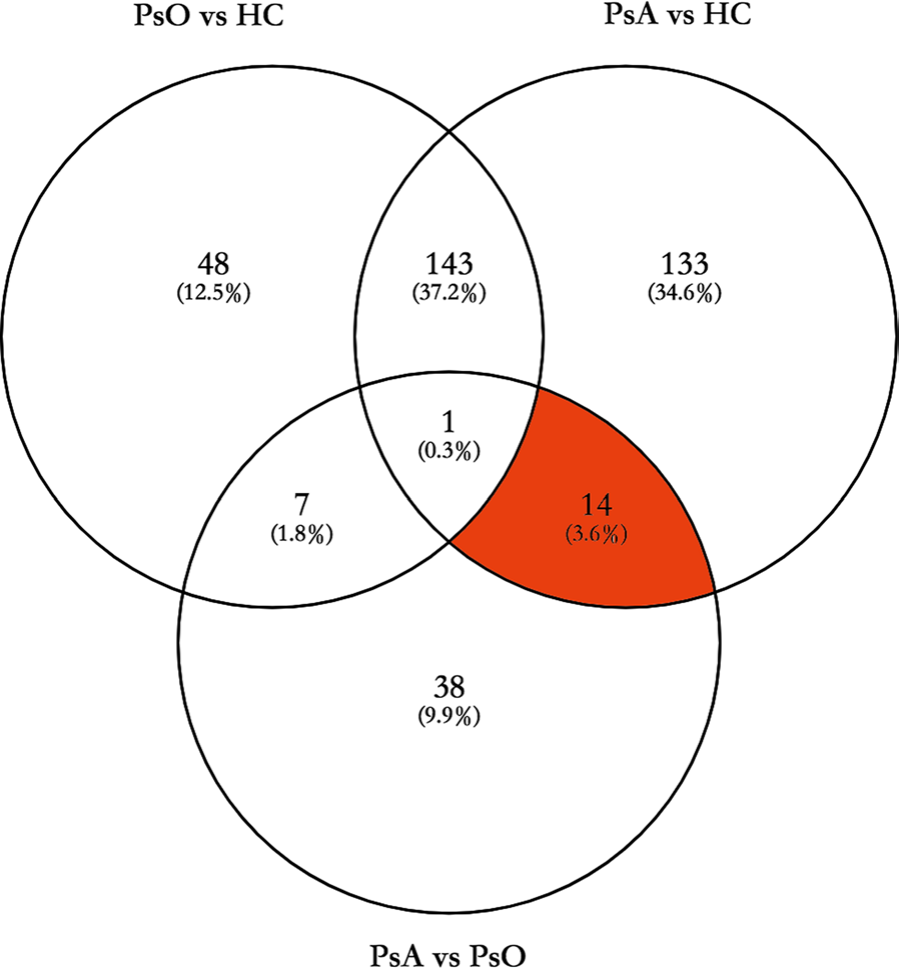
Venn diagram depicted the overlapping of significantly expressed proteins in the three datasets (PsO group versus HC group, PsA group versus HC group, PsA group versus PsO group). The red section included 14 proteins (protein SIRT2, NAA50, ARF6, ADPRHL2, SF3B6, SH3KBP1, UBA3, SCP2, RPS5, NUDT5, NCBP1, SYNE1, NDUFB7, HTATSF1) that met the criteria: (1) in the overlapping section of “PsA vs. HC” and “PsA vs. PsO”; (2) not in the section of “PsO vs. HC”

A total of 22,734 peptides and 3177 proteins were identified by iTRAQ and LC–MS/MS (false discovery rate, < 1%). After using *limma* for differential protein expression analysis, we identified 389 significantly changed proteins when comparing eight patients with psoriasis (PsO and PsA) and four healthy controls (HC). Similarly, we found 199, 291, and 60 significantly changed proteins when comparing PsO and HC, PsA and HC, and PsA and PsO, respectively. The list of all above significantly changed proteins including fold change and adjusted P values was uploaded as Additional file 4. By performing the Venn diagram of three significantly changed proteins group sets in Fig. 2, 14 proteins meeting the following criteria were marked in red: (1) in the overlapping section of “PsA vs. HC” or “PsA vs. PsO”; and (2) not in the section of “PsO vs. HC”. These were SIRT2, NAA50, ARF6, ADPRHL2, SF3B6, SH3KBP1, UBA3, SCP2, RPS5, NUDT5, NCBP1, SYNE1, NDUFB7, HTATSF1 (Table 1). Most of them were up-regulated in PsA (FC > 1), while RPS5 and HTATSF1 were down-regulated (FC > 1).

**Table 1.** 14 candidate predictive biomarkers for PsA

Proteins	Name	PsA/PsO		PsA/HC	
		Adj.p-value	FC	Adj.p-value	FC
SIRT2	Sirtuin-2	0.008	1.346	0.001	1.648
NAA50	N-alpha-acetyltransferase 50	0.027	1.531	0.001	1.735
ARF6	ADP-ribosylation factor 6	0.027	1.197	0.006	1.391
ADPRHL2	Poly(ADP-ribose) glycohydrolase ARH3	0.032	1.270	0.007	1.650
SF3B6	Splicing factor 3B subunit 6	0.027	1.359	0.008	1.597
SH3KBP1	SH3 domain-containing kinase-binding protein 1	0.049	1.267	0.011	1.466
UBA3	NEDD8-activating enzyme E1 catalytic subunit	0.030	1.254	0.017	1.333
SCP2	Non-specific lipid-transfer protein	0.015	1.225	0.018	1.273
RPS5	Ribosomal protein S5	0.008	0.645	0.024	0.689
NUDT5	ADP-sugar pyrophosphatase	0.030	1.208	0.029	1.317
NCBP1	Nuclear cap-binding protein subunit 1	0.049	1.337	0.030	1.370
SYNE1	Nesprin-1	0.018	1.344	0.035	1.324
NDUFB7	NADH dehydrogenase 1 beta subcomplex subunit 7	0.002	1.511	0.040	1.302
HTATSF1	HIV Tat-specific factor 1	0.018	0.738	0.048	0.787

*HC* healthy controls, *FC* fold change

**Fig. 3:**
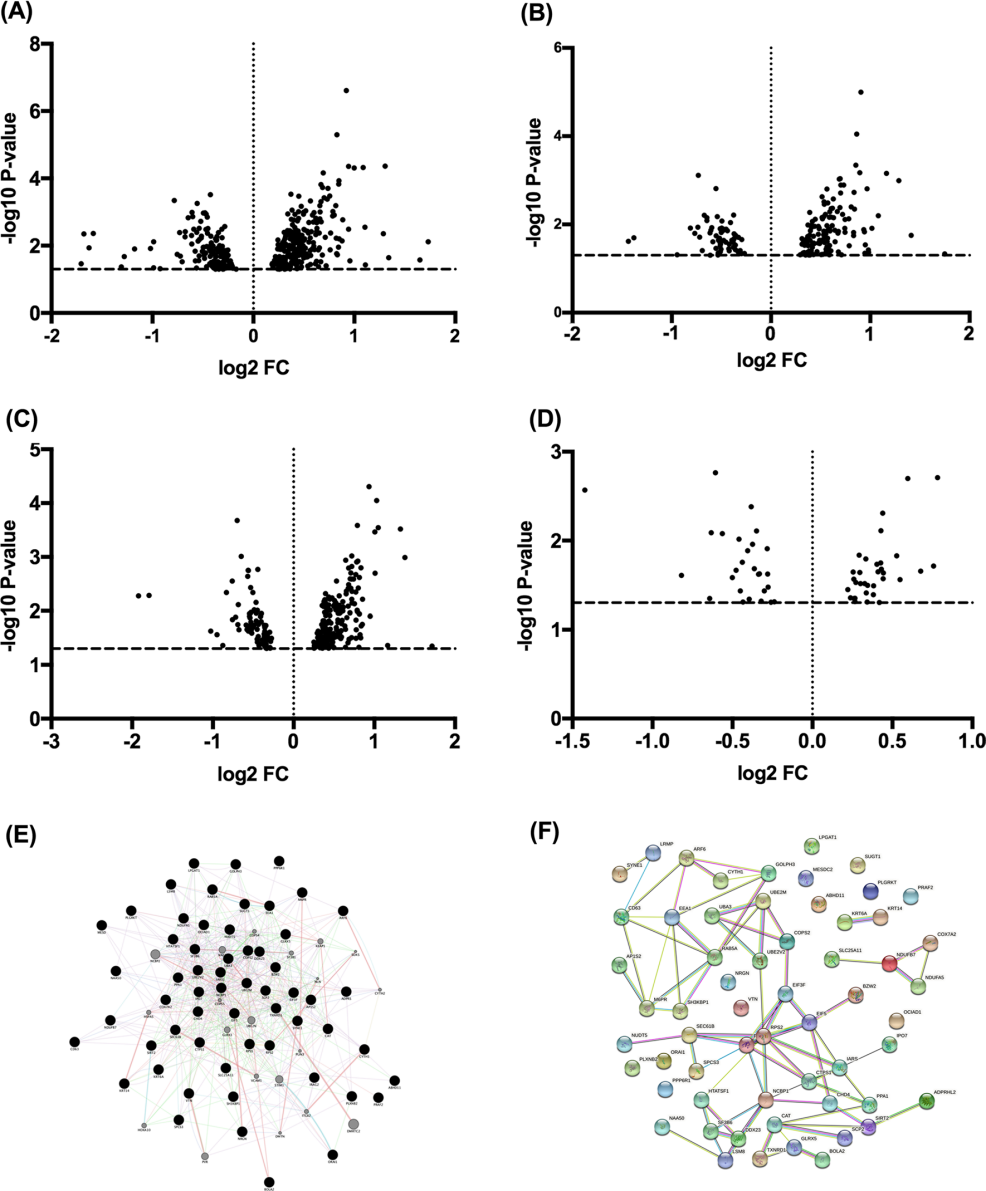
Volcano plot (fold change and significance) of protein expression changes in different group sets. **A** All patients with psoriasis versus HC group, **B** PsO group versus HC group, **C** PsA group versus HC group, and **D** PsA group versus PsO group. The fold change of all group sets were all log^2^ transformed and plotted against their -log^10^ transformed p-values. Each spot represents an identified protein. The threshold p = 0.05 is indicated as a horizontal line; the threshold of a ratio of fold change is indicated by vertical lines. The relationship of differentially expressed proteins in the PsA group versus PsO group was shown using **E** GeneMANIA and **F** STRING

Volcano plots of four significantly changed protein group sets were generated in Fig. 3A–D. In addition, we investigated the relationship of differentially expressed proteins in the PsA group versus the PsO group using GeneMania (Version v3.5.2, https://genemania.org) and STRING Version 11.0 (https://string-db.org/). (Fig. 3E, F). According to GeneMania, we identified several relationships among these proteins at the gene expression level, including co-expression (46.00%), physical interaction (33.06%), genetic interactions (10.05%), and co-localization (1.09%). In addition, according to STRING, 12 of the 60 proteins did not connect to any type of network (interaction score = 0.4), and none of the 12 proteins represent in the 14 candidate biomarkers. Forty-eight of the differentially expressed proteins were connected to networks by complex relationships.

### Bioinformatics analysis of the differentially expressed proteins

**Fig. 4:**
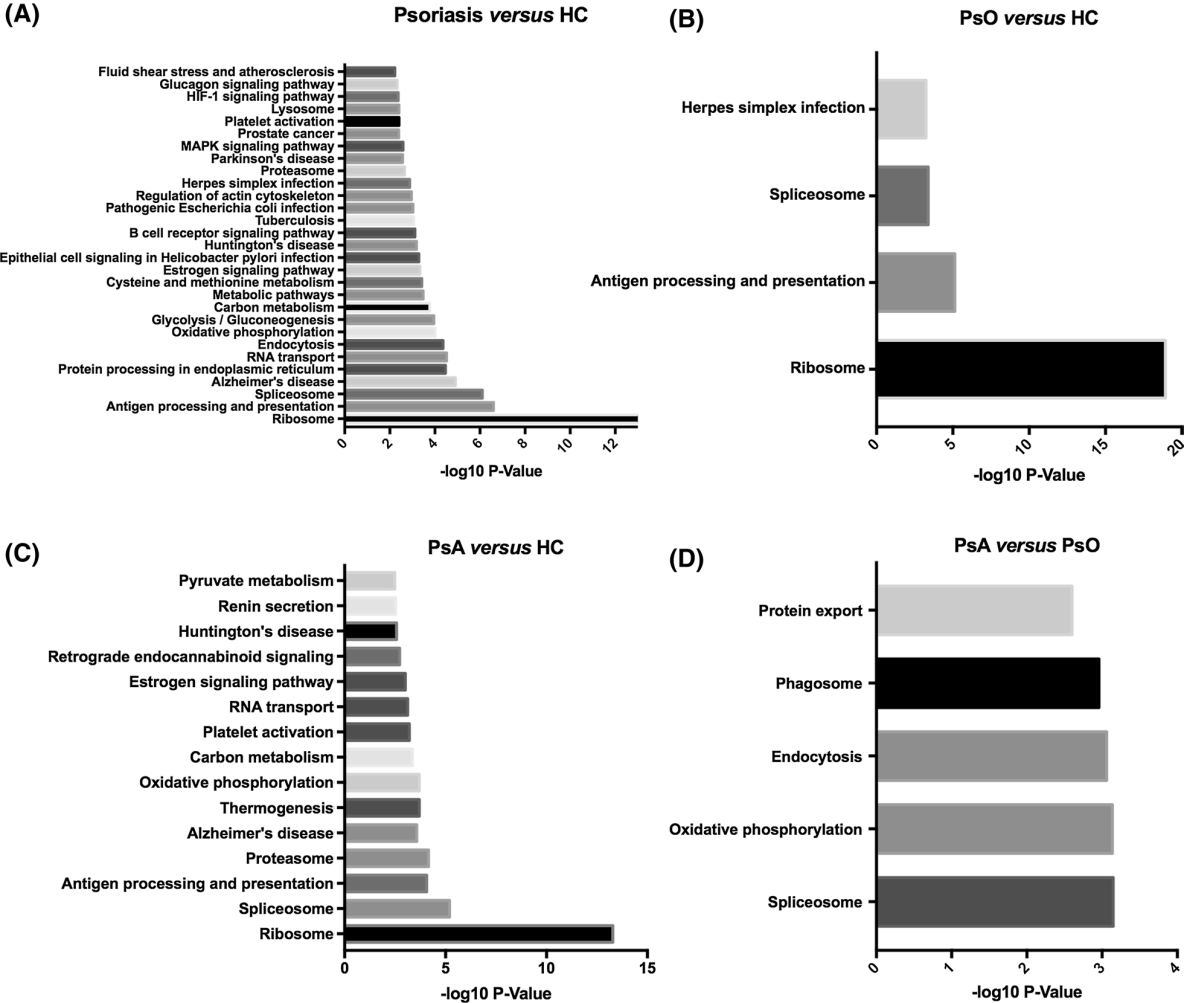
KEGG pathway analysis of protein expression changes in different group sets. **A** Psoriasis group versus HC group, **B** PsO group versus HC group, **C** PsA group versus HC group, and **D** PsA group versus PsO group. The p-values of each pathway were all − log^10^ transformed

To understand the biological characterization of differentially expressed proteins in four significantly changed proteins group sets, we performed KEGG pathway analysis using DAVID Bioinformatics Resources version 6.8 (Fig. 4).

Comparing all eight patients with psoriasis (PsO and PsA) and four healthy controls, top 10 highly enriched terms included ribosome, antigen processing and presentation, spliceosome, Alzheimer’s disease, RNA transport, protein processing in endoplasmic reticulum, endocytosis, oxidative phosphorylation, glycolysis/gluconeogenesis, carbon metabolism. In addition, KEGG pathway analysis also revealed several essential pathways, including spliceosome, oxidative phosphorylation, endocytosis, phagosome, and protein export when comparing patients with PsA and PsO.

### Western blotting validation of SIRT2 protein expression

**Fig. 5:**
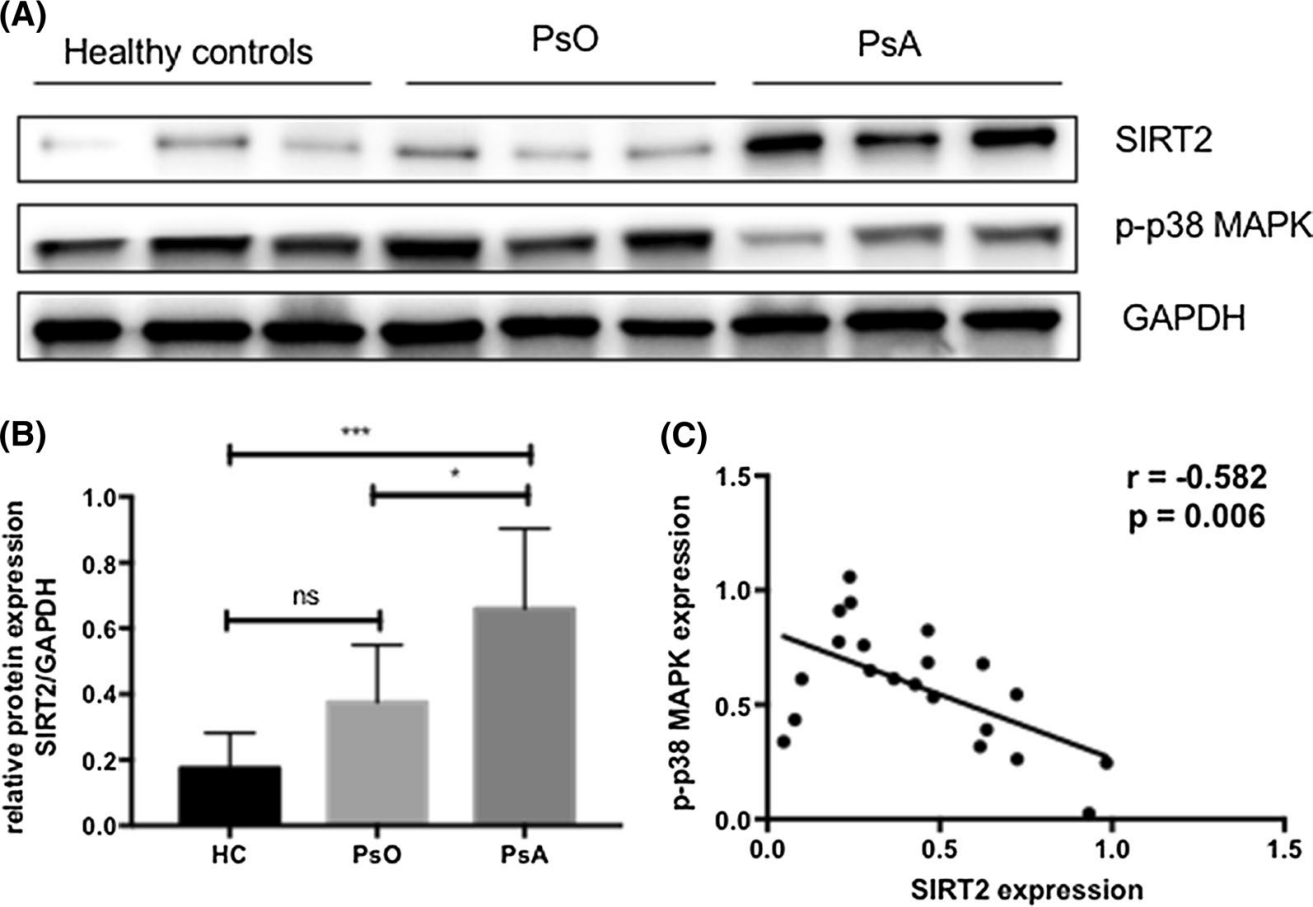
Western blotting validation of SIRT2 expression and its correlation with the phosphorylation of p38MAPK. **A** Representative western blot analysis of SIRT2, p-p38MAPK, and GAPDH as loading controls. **B** Relative SIRT2 protein expression was normalized to GAPDH. HC, healthy controls, n = 5; PsO, n = 8; PsA, n = 8. Data was expressed mean ± SD. *, P < 0.05; **, P < 0.01; ***, P < 0.001. **C** The correlation between SIRT2 and p-p38MAPK was shown in all 21 samples

To further investigate the expression of SIRT2 in PBMCs from patients with PsA and PsO, we compared the level of SIRT2 and the phosphorylation of p38 mitogen-activated protein kinase (p-p38MAPK) in 8 patients with PsO, 8 patients with PsA, and 5 healthy controls by western blot. Representative results were shown in Fig. 5A. As shown in Fig. 5B, SIRT2 expression was significantly higher in PsA compared with PsO (p = 0.024) and healthy controls (p = 0.001). Furthermore, the expression of SIRT2 was negatively correlated with the phosphorylation of p38MAPK (p = 0.006, r = − 0.582; Fig. 5C).

## Discussion

In this study, we adopted iTRAQ technology in combination with bioinformatics analysis and western blotting to investigate the proteome in PBMCs of patients with PsA and patients with PsO as well as healthy controls. PBMCs samples could be easily collected from patients by a minimally-invasive intervention, and our study represents the first proteomic research to use PBMCs as samples for exploring the different expressed proteins between patients with PsA and PsO. Taken differential proteins analysis performed by *limma* and Venn diagram results, we finally narrowed down the list of candidate biomarkers that distinguish PsA from PsO.

In the present study, proteomics analysis showed SIRT2 expression in PBMCs from PsA patients was significantly higher than PsO patients and healthy controls, and this finding was further validated by western blotting. SIRT2 is a member of the sirtuin protein family, which is localized primarily in the cytoplasm and can also shuttle to the nucleus [12]. SIRT2 has been implicated in the progress of inflammation in arthritis. A previous study demonstrated that SIRT2 mRNA expression is increased in active RA compared to inactive RA [13]. In addition, SIRT2 expression was found to be negatively correlated with the phosphorylation of p38MAPK in this study, which is consistent with the finding of Kim et al. [14] that SIRT2 suppressed the expression of pro-inflammatory cytokines and inhibited phosphorylation of p38MAPK in murine macrophages cells. However, SIRT2 was reported to promote the phosphorylation of p38MAPK in activated NK cells [15], kidney and tubular epithelial cells [16], and rabbit articular chondrocytes [17]. The contradictory effect of SIRT2 on the phosphorylation of p38MAPK needs to be further elucidated.

In the present study, KEGG pathway analysis of protein expression changes in the Ps (namely PsO + PsA) group versus HC group, PsA group versus HC group, and PsO group versus HC group all revealed differences in the ribosome pathway. Ribosomal protein S6, which is both a key mediator of mTOR function and coordinate regulator of ribosome function and biogenesis [18], is hyperactivated and abnormally phosphorylated in epidermal lesions of patients with psoriasis [19]. A quantitative proteomic analysis of psoriasis vulgaris also revealed that the differentially expressed proteins of skin lesions biopsies were mainly enriched in ribosomal pathway [20], supporting our finding in PBMCs.

The present study has several limitations. The major limitation is the small sample size and lack of control for differences between the sexes. While data from our small cohort generated distinctive and actionable data streams, it is clear that further validation of these preliminary findings in larger cohorts is necessary. Additionally, different PsA subtypes were not taken into consideration. Moll and Wright described five clinical subtypes of PsA in 1973 [21]. In this study, all patients with PsA fulfilled the CASPAR criteria for the diagnosis, and we decided not to classify PsA according to phenotypes for two reasons: (i) the phenotypes of PsA have been shown to change over time [22]; (ii) the enrolled PsA patients, consisting of the distal subtype, the oligoarticular subtype, the polyarticular subtype, and the spondylitis subtype, all showed the similar high expression level of SIRT2 in PBMCs.

## Conclusions

In conclusion, we developed iTRAQ-based quantitative proteomic approach to measure PBMCs protein expression profiling of PsA and PsO patients, and confirmed the higher expression of SIRT2 in PsA in an independent western blotting validation cohort. Although concerning a proof-of-concept study with limited sample size, these data provide a stepping-stone for follow-up research on the validation of the discovered biomarker candidates.

## Supplementary Information

**Additional file 1: Table S1.** Characteristics of patients for proteomics analysis.

**Additional file 2: Table S2.** Characteristics of patients for western blotting analysis.

**Additional file 3: Figure S1.** Raw Western blotting images for Fig.5 in manuscript. (A) Raw western blotting image for GAPDH (used as a loading control, 37 kDa); (B) Raw western blotting image for p-p38 (43 kDa); (C) Raw western blotting image for SIRT2 (43 kDa).

**Additional file 4.** The list of significantly changed proteins in the comparison of all psoriatic patients versus healthy controls, PsO group versus healthy controls, PsA group versus healthy controls, and PsA group versus PsO group, respectively.

**Additional file 5.** Sample processing of PBMCs for proteomics analysis.

## Data Availability

Reasonable requests for data will be made available for review.

## Abbreviations

ACOT9: Acyl-coenzyme A thioesterase 9
ADD3: Gamma-adducin
ADPRHL2: ADP-ribosylhydrolase like 2
ARF6: ADP-ribosylation factor 6
BSA: Body surface area
CASPAR: Classification criteria for psoriatic arthritis
COMP: Cartilage oligomeric matrix protein
COMT: Catechol O-methyltransferase
COX7A2: Cytochrome C oxidase subunit VIIa polypeptide 2
CXCL10: C–X–C motif chemokine 10
FASP: Filter assisted sample preparation
FDR: False discovery rate
HC: Healthy control
HLA: Human leukocyte antigen
HP: Haptoglobin
HPRP: High pH reverse phase fractionation
HTATSF1: HIV Tat-specific factor 1
IAA: Iodoacetamide
IL-17: Inter-leukin 17
ITGA5: Integrin alpha-5
iTRAQ: Isobaric tags for relative and absolute quantification
KEGG: Kyoto Encyclopedia of Genes and Genomes
LC–MS/MS: Liquid chromatography tandem mass spectrometry
MALDI-TOF MS: Matrix-assisted laser desorption/ionization time-of-flight mass spectrometry
MHC: Major Histocompatibility Complex
MMP: Matrix metalloproteinases
NAA50: *N*-Alpha-acetyltransferase 50
NAFLD: Non-alcoholic fatty liver disease
NDUFB7: NADH dehydrogenase [ubiquinone] 1 beta subcomplex subunit 7
NUDT5: ADP-sugar pyrophosphatase
OGDH: 2-Oxoglutarate dehydrogenase
PASI: Psoriasis Area Severity Index
PBMCs: Peripheral blood mononuclear cells
PsA: Psoriasis with psoriasis arthritis
PsO: Psoriasis without psoriasis arthritis
ROS: Reactive oxygen species
RP-HPLC: Reverse-phase high-performance liquid chromatography
RPLC: Reversed-phase liquid chromatography
RPS5: Ribosomal protein S5
SCP2: Non-specific lipid-transfer protein
SD: Standard deviation
SIRT2: NAD-dependent protein deacetylase sirtuin-2
TEAB: Tetraethyl-ammonium bromide
TLR: Toll-like receptor
TMSB4X: Thymosin beta-4
TNF-α: Tumor necrosis factor-α
Triple-TOF MS/MS: Triple time-of-flight mass spectrometry
TXNRD1: Thioredoxin reductase 1
UBA3: NEDD8-activating enzyme E1 catalytic subunit
UBE2V2: Ubiquitin-conjugating enzyme E2 variant 2
