# Supplementary material for: Identification of proteins associated with development of psoriatic arthritis in peripheral blood mononuclear cells: a quantitative iTRAQ-based proteomics study
Source: J Transl Med. 2021 Aug 3;19:331. doi: 10.1186/s12967-021-03006-x (PMC8336315; doi:10.1186/s12967-021-03006-x)
Supplement: Supplementary file 1 — Additional file 1: Table S1. Characteristics of patients for proteomics analysis. [file 12967_2021_3006_MOESM1_ESM.docx]

**Additional File 1**

**Table S1.** Characteristics of patients for proteomics analysis

|  | Sex | Age (y) | Duration of disease (y) | Diagnosis | PASI | BSA | Number of tender or swollen joints | Nail involvement | Dactylitis | RF | HBP | DM | Dyslipidemia | Smoking history |
| --- | --- | --- | --- | --- | --- | --- | --- | --- | --- | --- | --- | --- | --- | --- |
| 1 | M | 41 | 17 | PsO | 22.5 | 53.0 | N/A | No | N/A | N/A | No | No | No | Yes |
| 2 | M | 49 | 6 | PsO | 15.1 | 39.0 | N/A | No | N/A | N/A | No | No | No | Yes |
| 3 | M | 36 | 12 | PsO | 16.7 | 27.4 | N/A | No | N/A | N/A | No | No | Yes | No |
| 4 | M | 45 | 10 | PsO | 15.6 | 35.0 | N/A | No | N/A | N/A | No | No | No | Yes |
| 5 | F | 61 | 10 | PsA | 10.1 | 13.0 | 6 | Yes | Yes | (-) | No | No | No | No |
| 6 | F | 86 | 19 | PsA | 9.3 | 33.3 | 2 | Yes | No | (-) | Yes | No | No | No |
| 7 | M | 68 | 37 | PsA | 15.3 | 32.2 | 8 | Yes | Yes | (-) | No | Yes | No | Yes |
| 8 | F | 58 | 44 | PsA | 10.0 | 31.5 | 12 | Yes | Yes | (-) | Yes | No | Yes | No |

PASI, Psoriasis Area Severity Index; BSA, body surface area; HBP, hypertension; DM, diabetes mellitus; RF, rheumatoid factor
