# Supplementary material for: Identification of proteins associated with development of psoriatic arthritis in peripheral blood mononuclear cells: a quantitative iTRAQ-based proteomics study
Source: J Transl Med. 2021 Aug 3;19:331. doi: 10.1186/s12967-021-03006-x (PMC8336315; doi:10.1186/s12967-021-03006-x)
Supplement: Supplementary file 2 — Additional file 2: Table S2. Characteristics of patients for western blotting analysis. [file 12967_2021_3006_MOESM2_ESM.docx]

**Additional File 2**

Table S2. Characteristics of patients for western blotting analysis

|  | Sex | Age (y) | Duration of disease (y) | Diagnosis | PASI | BSA | Number of tender or swollen joints | Nail involvement | Dactylitis | RF | HBP | DM | Dyslipidemia | Smoking history |
| --- | --- | --- | --- | --- | --- | --- | --- | --- | --- | --- | --- | --- | --- | --- |
| 1 | F | 57 | 25 | PsO | 20 | 55 | N/A | No | N/A | N/A | No | Yes | Yes | No |
| 2 | M | 29 | 16 | PsO | 14 | 34.8 | N/A | No | N/A | N/A | No | No | No | No |
| 3 | M | 72 | 4 | PsO | 8.4 | 16.7 | N/A | No | N/A | N/A | No | No | No | Yes |
| 4 | M | 58 | 32 | PsO | 10.1 | 13.6 | N/A | Yes | N/A | N/A | Yes | Yes | No | No |
| 5 | M | 23 | 5 | PsO | 11.3 | 19.9 | N/A | No | N/A | N/A | No | No | No | No |
| 6 | M | 22 | 5 | PsO | 14 | 24 | N/A | No | N/A | N/A | No | No | No | Yes |
| 7 | M | 28 | 10 | PsO | 16.1 | 22.5 | N/A | No | N/A | N/A | No | No | No | No |
| 8 | M | 49 | 23 | PsO | 18.4 | 64 | N/A | No | N/A | N/A | Yes | No | Yes | Yes |
| 9 | M | 53 | 30 | PsA | 16.2 | 25.5 | 1 | Yes | No | (-) | No | No | Yes | No |
| 10 | F | 53 | 6 | PsA | 13.2 | 23.4 | 4 | Yes | No | (-) | No | No | Yes | No |
| 11 | M | 72 | 15 | PsA | 29.8 | 57.8 | 1 | Yes | No | (-) | Yes | No | No | Yes |
| 12 | M | 28 | 8 | PsA | 8.6 | 7.5 | 21 | Yes | Yes | (-) | No | No | No | Yes |
| 13 | M | 38 | 7 | PsA | 36.2 | 73.5 | 5 | Yes | Yes | (-) | No | No | Yes | No |
| 14 | F | 19 | 2 | PsA | 9.2 | 22 | 1 | No | No | (-) | Yes | No | No | No |
| 15 | M | 32 | 5 | PsA | 40.6 | 93.6 | 1 | Yes | No | (-) | No | No | No | No |
| 16 | F | 60 | 10 | PsA | 2.9 | 0.9 | 9 | Yes | Yes | (-) | Yes | No | No | No |

PASI, Psoriasis Area Severity Index; BSA, body surface area; HBP, hypertension; DM, diabetes mellitus; RF, rheumatoid factor.
