# Supplementary material for: Identification of proteins associated with development of psoriatic arthritis in peripheral blood mononuclear cells: a quantitative iTRAQ-based proteomics study
Source: J Transl Med. 2021 Aug 3;19:331. doi: 10.1186/s12967-021-03006-x (PMC8336315; doi:10.1186/s12967-021-03006-x)
Supplement: Supplementary file 3 — Additional file 3: Figure S1. Raw Western blotting images for Fig.5 in manuscript. (A) Raw western blotting image for GAPDH (used as a loading control, 37 kDa); (B) Raw western blotting image for p-p38 (43 kDa); (C) Raw western blotting image for SIRT2 (43 kDa). [file 12967_2021_3006_MOESM3_ESM.pdf]

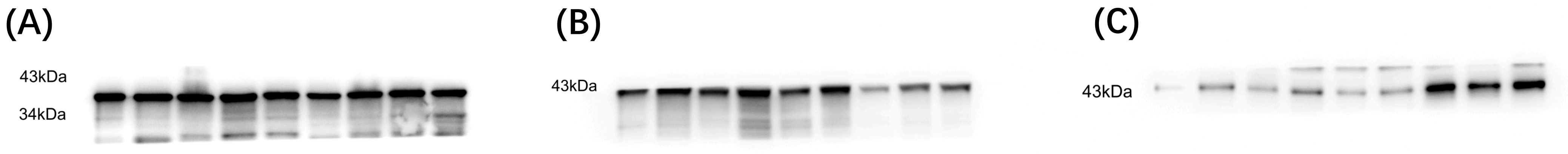

Figure. Raw Western blotting images for Fig.5. (A) Raw western blotting image for GAPDH (used as a loading control, 37kDa); (B) Raw western blotting image for p-p38 (43kDa); (C) Raw western blotting image for SIRT2 (43kDa).
