# Supplementary material for: Identification of proteins associated with development of psoriatic arthritis in peripheral blood mononuclear cells: a quantitative iTRAQ-based proteomics study
Source: J Transl Med. 2021 Aug 3;19:331. doi: 10.1186/s12967-021-03006-x (PMC8336315; doi:10.1186/s12967-021-03006-x)
Supplement: Supplementary file 5 — Additional file 5. Sample processing of PBMCs for proteomics analysis. [file 12967_2021_3006_MOESM5_ESM.docx]

**Additional File: Sample processing of PBMCs samples for proteomics analysis**

**Proteomic preparation and iTRAQ labeling**

After recovering, 12 PBMCs samples for proteomics analysis were lysed and homogenized with 4% SDS buffer which contained 1 mM dithiothreitol, 1% (v/v) protease inhibitor cocktail. An equal amount of protein (100 μg) from each sample was prepared using Filter Assisted Sample Preparation (FASP) procedure [11]. Briefly, each sample was transferred to a 30-kDa filter. After centrifugation at 10,000 ×g for 30 min at 20°C, 150 μL of urea buffer (8 M urea, 0.1 M Tris-HCl, pH 8.5) was added and followed by another centrifugation at 10,000 ×g for 40 min. 100 μL of iodoacetamide (IAA, 50 mM) were mixed with the samples and incubated for 20 min at room temperature, dark area. Next, IAA was removed by centrifugation at 10,000 g for 30 min. Then, samples were dissolved in 150 μL tetraethyl-ammonium bromide (TEAB, 0.1 M) and centrifuged at 10,000 g for 40 min. This step was repeated four times. Finally, samples were then digested with trypsin (1:50) at 37°C for 16 h (overnight) and centrifuged at 12,000 rpm for 20 min. To quantify 12 samples, 2 batches of 8-plex iTRAQ labeling experiment were performed with 8-plex iTRAQ reagent (Applied Biosystems, Foster City, CA, USA) according to the manufacturer's protocol. 12 PBMC samples were grouped and individually labeled with iTRAQ-114, 115, 116, 117, while mixtures of all 12 samples were labeled with iTRAQ-113 to serve as internal standard controls (Table S1).

**TableS1.** iTRAQ-labelling strategy for all samples

| Group | Sample | iTRAQ labeling |
| --- | --- | --- |
| HC | MIX | 113 |
|  | HC1 | 114 |
|  | HC2 | 115 |
|  | HC3 | 116 |
|  | HC4 | 117 |
| PsO | MIX | 113 |
|  | PsO1 | 114 |
|  | PsO2 | 115 |
|  | PsO3 | 116 |
|  | PsO4 | 117 |
| PsA | MIX | 113 |
|  | PsA1 | 114 |
|  | PsA2 | 115 |
|  | PsA3 | 116 |
|  | PsA4 | 117 |

**High pH reverse phase fractionation (HPRP)**

iTRAQ-labeled peptides was resuspended using buffer A. Buffer A consisted of 10 mM ammonium formate, and buffer B consisted of 10 mM ammonium formate with 90% acetonitrile; both buffers were adjusted to pH 10 with ammonium hydroxide. A CBS-B programmed automatic multifunction fraction collecting instrument was used to collect eluted peptides. A total of 28 fractions were collected, and then concatenated to 14 (pooling equal interval reversed-phase liquid chromatography (RPLC) fractions). The fractions were dried for nano liquid chromatography-tandem mass spectrometry (LC-MS/MS) analysis.

**LC-MS/MS analysis**

The reverse-phase high-performance liquid chromatography (RP-HPLC) separation was achieved on the Easy nano-LC system (Thermo Fisher Scientific) using a self-packed column (75 µm × 150 mm; 3 µm ReproSil-Pur C18 beads, 120 Å, Dr. Maisch GmbH, Ammerbuch, Germany) at a flow rate of 300 nL/min. The column temperature was maintained at 25 °C and the sample injection volume was 4 μL. The mobile phase A consisted of 0.1% formic acid, and B consisted of 0.1% formic acid in acetonitrile. The peptides were eluted using a gradient (2–90% mobile phase B) over 90 min period. Data was obtained using a nano-ESI Orbitrap Elite mass spectrometer (Thermo Fisher Scientific). The mass spectrometer was operated in data-dependent mode with each full MS scan (m/z 300–1500) followed by MS/MS for the 12 most intense ions with the parameters: precursor ion charge, ≥ +2; precursor ion isolation window, 2 Da; first mass, 80; normalized collision energy of HCD, 38; Dynamic Exclusion™, 30 s. The full mass and the subsequent MS/MS analyses were scanned in the Orbitrap analyzer with R = 60,000 and R = 15,000, respectively. The peptides were filtered to an FDR of lower than 1%. Only proteins identified with at least one single peptide, high confidence (FDR < 0.01 at peptide and protein level) and present in two or three biological replicates were considered. To correct for multiple comparisons, the FDR was controlled with the Benjamini–Hochberg procedure.
